# Inequalities in public water supply fluoridation in Brazil: An ecological study

**DOI:** 10.1186/1472-6831-8-9

**Published:** 2008-04-10

**Authors:** Marilisa CL Gabardo, Wander J da Silva, Marcia Olandoski, Simone T Moysés, Samuel J Moysés

**Affiliations:** 1 Pontifical Catholic University of Paraná, Curitiba, Brazil; 2 Faculty of Dentistry of Piracicaba, UNICAMP, Piracicaba, Brazil

## Abstract

**Background:**

The literature is scarce on the social and geographic inequalities in the access to and implementation of the fluoridation of public water supplies. This study adds knowledge to the Brazilian experience of the chronic privation of water and wastewater policies, access to potable water and fluoridation in the country. Thus, the aim of this study was to verify possible inequalities in the population's access to fluoridated drinking water in 246 Brazilian municipalities.

**Methods:**

The information on the process of water fluoridation in the municipalities and in the macro region in which each municipality is located was obtained from the national epidemiological survey which was concluded in 2003. The data relating to the human development index at municipal level (HDI-M) and access to mains water came from the Brazilian Human Development Atlas, whilst the size of the population was obtained from a governmental source. The Fisher exact test (*P *< 0.05) was employed to identify significant associations between the explanatory variables and their ability to predict the principal outcomes of interest to this study, namely the presence or absence of the water fluoridation process in the municipalities as well as the length of time during which this measure has been implemented. Linear regression was used to observe the associations between the relevant variables in a multivariate environment.

**Results:**

The results clearly showed that there is a relationship between municipalities with larger populations, located in more socio-economically advantaged regions and with better HDI-M, and where fluoridation is both present and has been implemented for a longer period of time (started before 1990).

**Conclusion:**

The findings suggest that the aim of treating water with fluoride may not be being adequately achieved, requiring more effective strategies so that access to this measure can be expanded equitably.

## Background

In global health terms, approaching the subject of inequalities in access to public health policies is a classic issue and one that is always current [1,2]. The literature suggests that populations and individuals more socially advantaged would appear to be benefited by measures that potentially influence the determining factors of health, reduce the risk of disease and control harm, since they are the ones that are primarily reached by public policies. Regarding oral health, studies on human populations demonstrate that the number of teeth affected by caries tends to be fewer in communities that have access to fluoridated water than in areas not covered by this measure [3-5].

Indeed, the fluoridation of public water supplies is considered to have been one of the ten most important public health measures in the last century [6,7] and is recommended by international health organizations [8,9]. Systematic reviews of the literature on fluoridation prove its beneficial and safe effects on human health [10], and its impact on the reduction of tooth decay owing to fluoridation has been observed in Brazil and in other regions in the world [3-5,11,12]. This hypothesis has also been tested by some Brazilian studies in relation to the process of fluoridation, and it has been possible to observe that the presence of fluoridated water occurs in a considerably heterogeneous manner, maintaining a positive association with better social, economic and demographic indicators [13,14].

There is increasing evidence in Brazil that the experience of tooth decay occurs in parallel to the social differences among the population [15-23]. This is partially confirmed by the data made available through the most recent national epidemiological survey [24]. The country's North and Northeast macro regions have greater average prevalence of tooth decay in the population of 12 year-olds, and an index of decayed, missing or filled teeth (DMF-T), of 3.13 and 3.19, respectively. These averages are higher than those for the South and Southeast macro regions and are higher than the overall Brazilian average of 2.78. The preceding direct observation does not account for the entire problem in relation to the access of economically less advantaged populations to fluoridation. Currently, the discussion on the relationship between fluoridation and socio-economic inequalities that probably impact on oral health appears to have gained new strength. Research findings from the 1980s [25,26] suggested that children who lived in areas of greater social privation, but with access to water treated with fluoride, had better oral health than those from a similar social situation without access to fluoridated water, or than those from a higher social class but who lived in communities without fluoridated water [25,26]. In the 1990s, new findings brought the knowledge that individuals with low socio-economic power and who do not have access to other sources of fluoride or to dental care services, would be considerably benefited if they had access to water treated with fluoride [27,28]. 1990 was the baseline for Brazil's present health system. In that year the Public Health Organic Laws Nos. 8080 and 8142 were established, bringing repercussions for the process of expanding water fluoridation in Brazil [29].

In the light of the above, the general purpose of this study was to verify how social and geographic inequalities are interfering with Brazilian public water supply fluoridation policy.

## Methods

In this study, a quantitative analysis was carried out using an ecological research design. This design was considered appropriate for the objectives proposed, in the light of the interest in verifying the impacts of interventions on populations, such as new programmes, legislation or policies [30]. The study was done using a secondary database from the national epidemiological survey on oral health called *SB Brasil Project *[24]. The population of the present research was comprised of the 250 municipalities studied in the above survey.

The independent variables were: the Municipal Human Development Index (HDI-M) for the year 2000, the percentage of access to mains water and the size of the population also for the 2000 year, as well as the macro region to which each municipality belonged. The size of the municipal population was obtained from a governmental source [31].

The HDI and the percentages of access to mains water were obtained from version 1.0.0 of the Brazilian Human Development Atlas, produced by the United Nations Development Programme (UNDP), which provides data for the years 1991 and 2000 [32]. When the relation between the social determinants of quality of life, and general and oral health outcomes are analyzed, it is necessary to consider the determining factors of inequalities in health [14,17,20-22,33,34]. The Human Development Index (HDI) is a powerful instrument created by the United Nations in the beginning of the 1990's (32), that takes into account the multidimensionality of differences in living conditions. The HDI relates to: (a) the population longevity, expressed by life expectancy; (b) adult literacy rate, composed of two variables: the adult literacy rate and combined rate of enrollment in three levels of instruction; and (c) income or Gross National Product (GNP) per capita, expressed in US dollars and adjusted to reflect the purchasing power parity (PPP) among citizen from several countries. This index ranges from 0 to 1, ranking the places into the levels: low (0 = IDH < 0.5), medium (0.5 = IDH < 0.8) and high human development (0.8 = IDH ≤ 1). When it is related to the municipal level, an "M" is added, and this index (HDI-M) is also widely used in studies on quality of life and socioeconomic conditions of populations.

The macro region variable and its dependent variables – water fluoridation in the municipalities involved (presence or absence of fluoridation) and the year in which this measure was implemented – were taken from the *SB Brasil *project's national database [24]. Municipalities that did not have complete information pertinent to the study's variables were excluded. As such the final sample contained 246 municipalities.

The Student's t test was performed to determine whether there was a significant difference between the average HDI-M values for the years 1991 and 2000. Pearson's correlation was used to analyse whether the HDI-M values for these years present a correlated variation, that is to say, whether those with a better classification in 1991 tended to maintain this level in 2000, and vice versa.

As the HDI-M (and sub-indices), percentage access to mains water and population size variables did not present normal distribution they were dichotomized using a median split. The use of the ANOVA and Newman-Keuls tests enabled the macro regions to be dichotomized into: North-Northeast and South-Southeast-Mid West. The dependant variable relating to fluoridation was categorized into: "fluoridation begun by the year 1990"; "begun after 1990"; and "absence of fluoridation". Bivariate analysis was then carried out (Fisher's Exact Test with *P *< 0.05), to identify associations between the explanatory variables and their ability to predict the outcome of interest, namely "fluoridation." Also, the association between the HDI-M and percentage access to mains water was verified.

Multivariate analysis was performed. The technique chosen was linear regression and it was used in two ways: simple linear regression was used when the issue was the outcome variable in its dichotomic form, i.e. the presence or absence of fluoridation. When the analysis was conceived using the outcome variable with three temporal categories (fluoridation begun by 1990, fluoridation begun after 1990 and absence of fluoridation), ordinal regression was opted for and which, in English, is referred to by the acronym PLUM (*Polytomous Logit Universal Models*).

Both regressions were performed using a significance level of *P *< 0.05, and the aggregated form and the sub-indices of the HDI-M were not included together in the regression, since this could result in a loss of the explanatory power due to the fact of the HDI-M arising from a prior aggregation of other components.

## Results

The Student's t test showed significant difference (*P *< 0.0001) between HDI-M values for the years 1991 and 2000 of 0.647 and 0.727, respectively (sd = 0.02). Pearson's correlation indicated that the growth in the HDI-M for these years was highly correlated (r = 0.969, *P *< 0.0001). Consequently it was only possible to work with the year 2000 data.

Of the 246 municipalities studied, 113 (45.9%) had water supply fluoridation, whilst 133 (54.1%) did not have this measure. Table 1 shows the percentage of fluoridation in the different macro regions studied.

**Table 1:** Percentage of fluoridation in Brazil's five macro regions.

Macro region	Fluoridation			
	
	With		Without	
	
	n	%	n	%
North	2	4.1	47	95.9
Northeast	8	16.3	41	83.7
Mid West	27	54.0	23	46.0
South	44	88.0	6	12.0
Southeast	32	66.7	16	33.3

**TOTAL**	113	—	133	—

**Source: ***SB Brasil *Project .^20^

The significant relationship (P < 0.0001) between the HDI-M and percentage access to mains water can be seen in Table 2 It shows the evident association between municipalities with a higher HDI-M rating and higher mains water percentage. However, in linear regression, the HDI-M and percentage access to mains water can not show so significant associaton whilst the size of the population and macro region seem to present some positive influence as to the coverage of fluoridation in the municipalities.

**Table 2:** Association between the HDI-M and percentage access to mains water in the year 2000.

Variable	n	Percentage access to mains water > 84.24% n = 123 n(%)	Percentage access to mains water ≤ 84.24% n = 123 n(%)	*P*-value* (bivariate)
HDI-M				
> 0.743	121	103 (85.1)	18 (14.9)	< 0.0001
≤ 0.743	125	20 (16.0)	105 (84.0)	

(*) Fisher's Exact Test (*P *< 0.05).

Table 3 shows the results of the bivariate and multivariate analyses for the fluoridation process outcome. In the same way as happened previously, in the bivariate analysis municipalities with better HDI-M, larger populations and which are located in the South, Southeast and Mid West regions implemented the measure prior to 1990, and this reinforces the geographic inequality in the distribution of the measure.

**Table 3:** Bivariate and multivariate analyses according to the time of the variable fluoridation in the 246 municipalities studied.

Variable	n	Fluoridation before 1990 n = 72 n(%)	Fluoridation after 1990 n = 41 n(%)	Without fluoridation n = 133 n(%)	*P*-value* (bivariate)	*P*-value** (multivariate)
HDI-M						
> 0.743	121	54 (44.6)	28 (23.1)	39 (32.2)	< 0.0001	0.416
≤ 0.743	125	18 (14.4)	13 (10.4)	94 (75.2)		
Percentage access to mains water						
> 84.24%	123	59 (48.0)	26 (21.1)	38 (30.9)	< 0.0001	0.213
≥ 84.24%	123	13 (10.6)	15 (12.2)	95 (77.2)		
Population size						
> 24440 inhabitants	122	50 (41.0)	21 (17.2)	51 (41.8)	< 0.0001	< 0.0001
≤ 24440 inhabitants	124	22 (17.7)	20 (16.1)	82 (66.1)		
Macro region South/Southeast	148	69 (46.6)	34 (23.0)	45 (30.4)	< 0.0001	< 0.0001
Mid West and North/Northeast	98	3 (3.1)	7 (7.1)	88 (89.8)		

(*) Fisher's Exact Test (*P *< 0.05)

(**) Linear regression (*P *< 0.05)

The location of municipalities in socio-economically less developed regions is more associated with the absence of fluoridation (*P *< 0.001). With regard to the ordinal regression analysis, the population size and the macro region maintained a difference in relation to the fluoridation process, which indicates that these variables are independently associated with the fluoridation process (*P *< 0.0001).

## Discussion

This study includes data on 246 municipalities which took part in the national epidemiological survey and which are considered to be representative of the country's population [24]. The results show that municipalities located in macro regions with better socio-economic development are significant associated with the presence of fluoridation as well as with the early implementation of this measure, i.e. by the year 1990. The implementation of the National Health System (NHS) with effect from 1990 does not appear to have affected this pre-1990 tendency. This indicates that the public policy on oral health should seek to align itself with other public policies of a more equitable nature, placing particular emphasis on the issue of unjust and avoidable inequalities.

The relationship between social indicators and caries prevalence has been extensively researched [17-23,27]. In Brazil, the North and Northeast regions have greater average prevalence of tooth decay in the population of 12 year-olds, and an index of decayed, lost or filled teeth (DMF-T), of 3.13 and 3.19, respectively. These averages are higher than those for the South and Southeast regions and are higher than the Brazilian average of 2.78. The former regions also have a larger number of municipalities without fluoridated water (89.8%), and this coincides with areas of greater social privation [24].

The present study has sought to add knowledge regarding the influence of some variables relating to living conditions on the process of the fluoridation of the public water supply, since great importance is attributed to this measure in caries reduction all over the world [10-12].

With regard to the methodology used in this study, the ecological design is justified in the light of the objective of evaluating the impact of a public policy, such as the fluoridation of the water supply, on population aggregates [30], without the intention of inferences being made on an individual level. The main topic of interest was the verification of the association between fluoridation outputs and the explanatory variables of HDI, population size, macro region and percentage access to mains water.

Care was taken to perform a correlation to check the stability of the HDI-M variable between 1991 and 2000, and consequently it was possible to work with the 2000 data. This index, despite criticisms owing to its being an aggregated representation [35], was chosen for use in this study as it provides multidimensional characteristics of living conditions. Its sub-indices on income, education and longevity were also included in the analyses, with the aim of identifying whether they would reveal an association with the outcomes of interest. However, the HDI-M did not show itself to be significant in the multivariate analysis in any of its forms.

The observations presented corroborate the inverse care law, which states that the availability and the proper provision of health services vary inversely to the needs of the population [1]. As such, health resources are subject to market influences and primarily meet the needs of socio-economically more advantaged populations. In addition, the first to be reached by new health interventions are those people in a better situation and this in turn contributes towards the growing inequality in health services [2].

Previous alerts are of particular relevance, taking into consideration that in countries like the United States, England and New Zealand, water fluoridation is considered to be a measure capable of reducing not only the prevalence and the severity of dental caries, but also the disparities between populations with different socio-economic conditions [36]. This must be plausible for Brazil as well.

Research into the association between the presence or absence of fluoridation, how long it has been implemented for, and socio-economic indicators, has produced results that are consistent with those of this study, such as the strong association between human development and the presence of fluoridation, with loss of statistical significance in the presence of other variables. Similarities were also found with regard to the measure being implemented later in less developed municipalities [13].

Another study, also based on the results of the SB Brasil Project [24], assessed the role of fluoridation in the reduction of oral health inequalities [14]. The findings of the study suggest that fluoridation benefits more, and earlier, municipalities with better socio-economic indicators, in the same way that a greater number of such municipalities are also connected to the water supply mains [14]. The findings of the present study are also in agreement with this, since municipalities with a better HDI-M have more access to mains water.

The findings of this study indicate that the size of the population and location within potentially "richer" macro regions are significantly associated with the presence of fluoridation, whilst the HDI-M, in any of its forms, did not show itself to be significant in the multivariate analysis. This may suggest that if the HDI-M is taken in isolation, it loses its explanatory power. That is to say, the context of the macro region and its structural level of development may result in municipalities with better HDIs, but located in poorer regions, not having fluoridation, whilst municipalities with worse HDIs located in more prosperous regions may have implemented fluoridation.

With regard to the size of the population, a statistically significant association with fluoridation was observed in the analyses undertaken, with evidence that this measure is concentrated in larger municipalities. The literature reveals that smaller municipalities, as a result of their per capita income, are not able to implant this measure adequately, since a measure that precedes fluoridation is the implantation of water treatment systems [37]. At the centre of this discussion, although it strays from the scope of this study, is the issue of the historical and growing tendency of the provision of potable water as a commodity, thus becoming a preferential target for privatization proposals. This is contrary to the fact that, regardless of their size, all municipalities raise funds that should be destined towards fluoridation [38].

There are indications that the expansion of the fluoridation coverage among the population depends on federal government incentives and not on the privatization of the water and wastewater sector, as well as on a culture among both the population and institutions that favours the implementation of this measure [39].

In a recent case study in the State of São Paulo, Brazil, focusing the costs of public water supply fluoridation between 1985 and 2003, the conclusion was reached that justifying the non-implementation of this measure due to the high cost is a fallacy. The cumulative cost in the period under study was R$ 1.44 (US$ 0.97) per capita. The estimated annual per capita cost for 2003 was R$ 0.084 (US$ 0.028). The findings make it clear that the amount is insignificant when compared to the benefits provided by fluoridation, in terms of the improvement in the population's quality of life, and the reduction in demand for oral and dental treatment [40].

It is appropriate to discuss the social and geographic inequality to which the Brazilian population is subjected with regard to fluoridation, despite it being guaranteed by law [41]. Another cause for concern is that equality is one of the basic principles of the National Health System (NHS) [29]. Despite Brazilian health legislation having advanced in terms of equality since the 1990s there is still profound inequality between regions and social groups. Concentration of resources occurs in regions that are less benefited by economic and social policies, thus contributing towards the increase of social inequalities that are reflected in health services. As such, the conclusion is reached that the public sector should ensure that unjust and avoidable factors present in health services be progressively reduced, and that the population be provided with all necessary health care and services in an equitable manner [42,43]. It should also be stated that the health services are responsible not only for offering care but also for making available the means necessary to alter the conditions that create, maintain or increase poverty.

## Conclusion

Within the limitations that are proper to an ecological study, whereby observations gathered on an aggregated level cannot be inferred on an individual level, the conclusion is reached that it is important to give heed to the need for strategic public policies that ensure the expansion of water fluoridation to places that are less socio-economically advantaged so as to contribute towards the reduction in health inequalities.

## Pre-publication history

The pre-publication history for this paper can be accessed here:


